# Supplementary material for: Bayesian brain mapping: A population-informed framework for personalized functional network topography and connectivity
Source: Imaging Neurosci (Camb). 2026 Oct 27;4:IMAG.a.1411. doi: 10.1162/IMAG.a.1411 (PMC13716283; doi:10.1162/IMAG.a.1411)
Supplement: Supplementary file 1 [file IMAG.a.1411_supp.pdf]

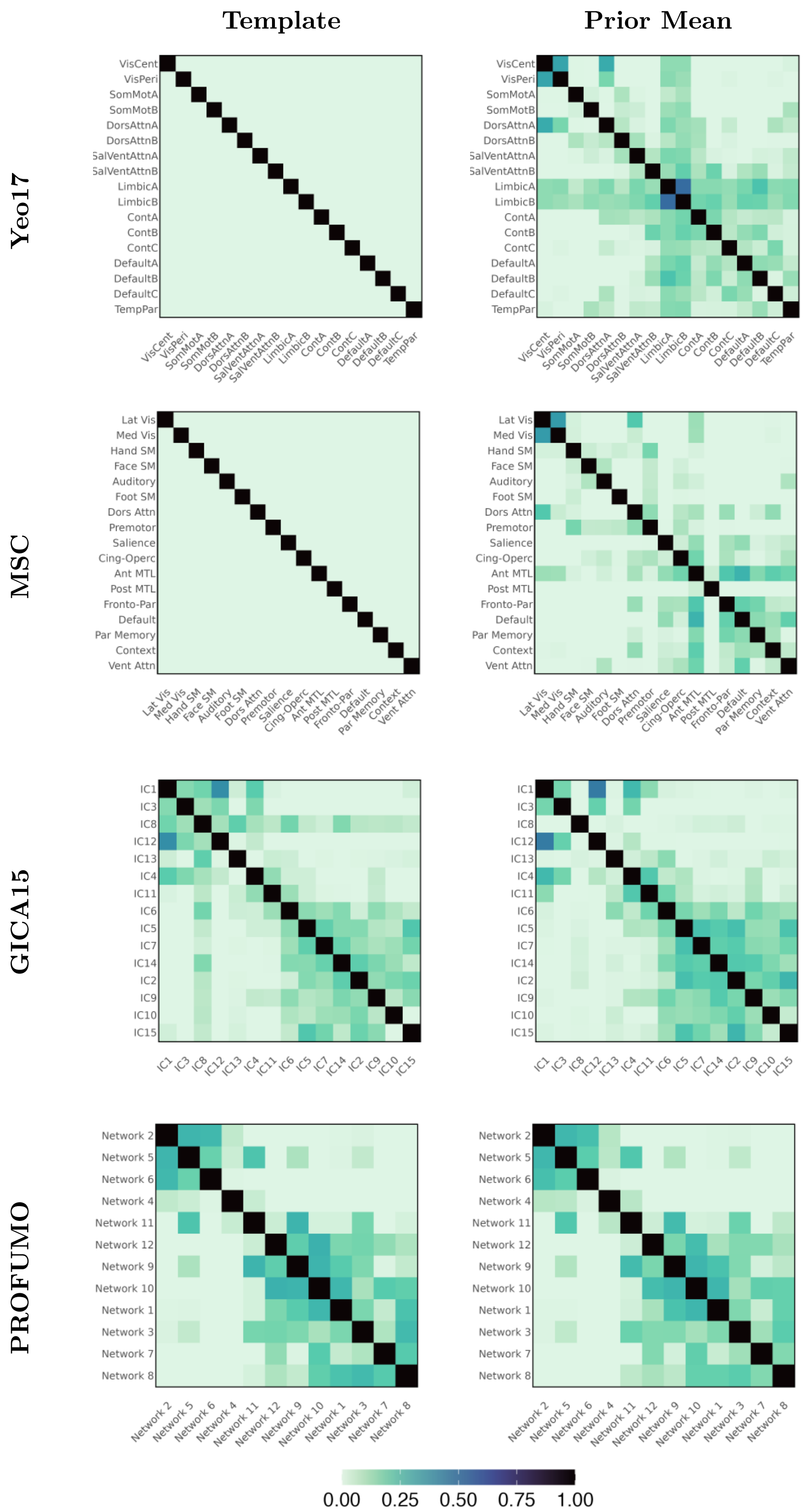

Figure S.1: Dice overlap matrices comparing each template with its prior mean. Yeo17 and MSC used hard labels; GICA and PROFUMO maps were thresholded at $|z| \geq 2$ before computing Dice. For MSC, GICA and PROFUMO, the networks were matched to the Yeo17 networks based on spatial overlap, so that the network ordering of the matrices is approximately consistent across templates.

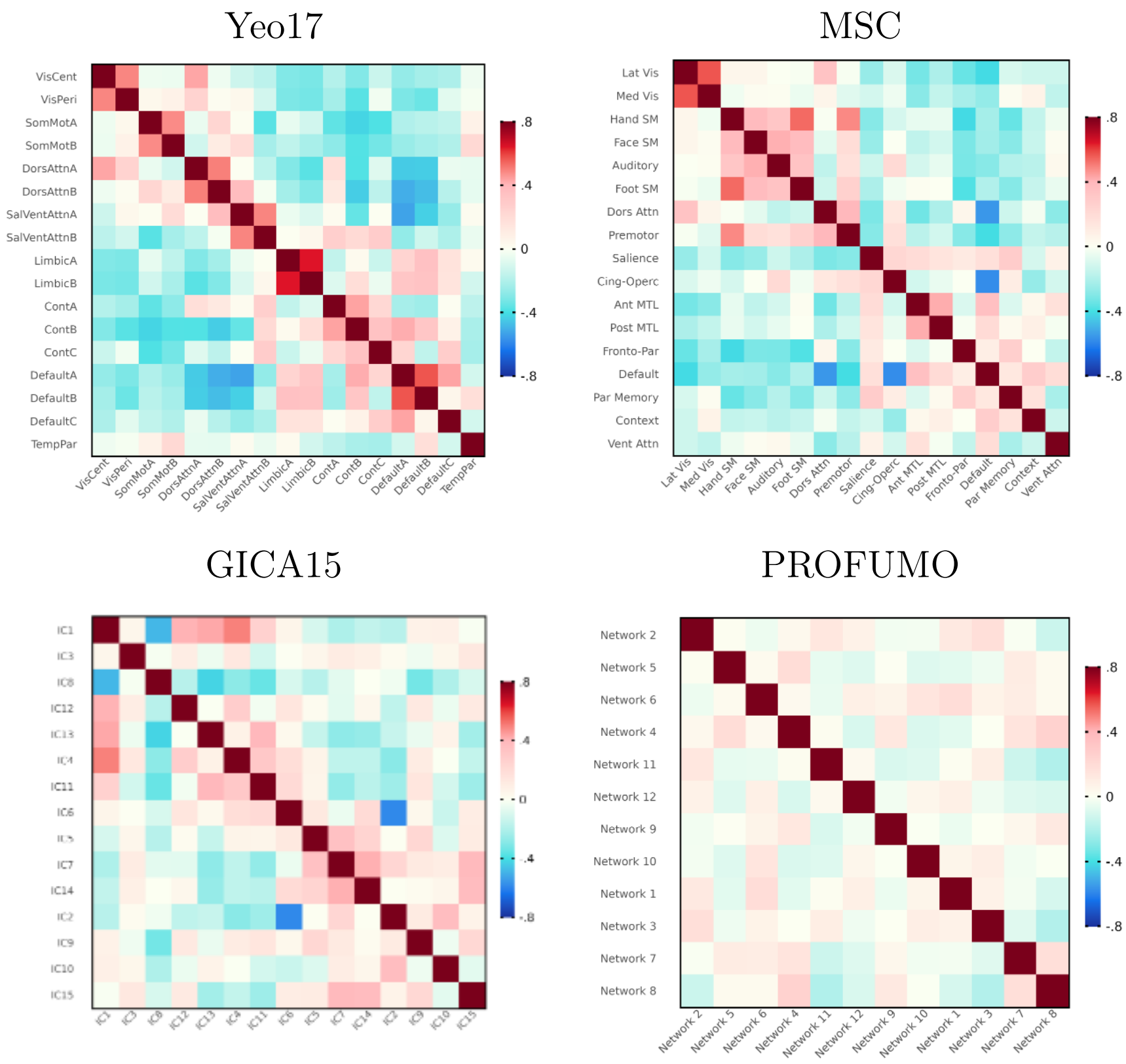

Figure S.2: Functional connectivity (FC) priors for various templates. The empirical element-wise population mean of FC across the prior training sample is shown. The empirical population mean and variance are not used directly in the BBM model. Rather, the inverse-Wishart or permuted Cholesky prior is built based on the FC training samples.

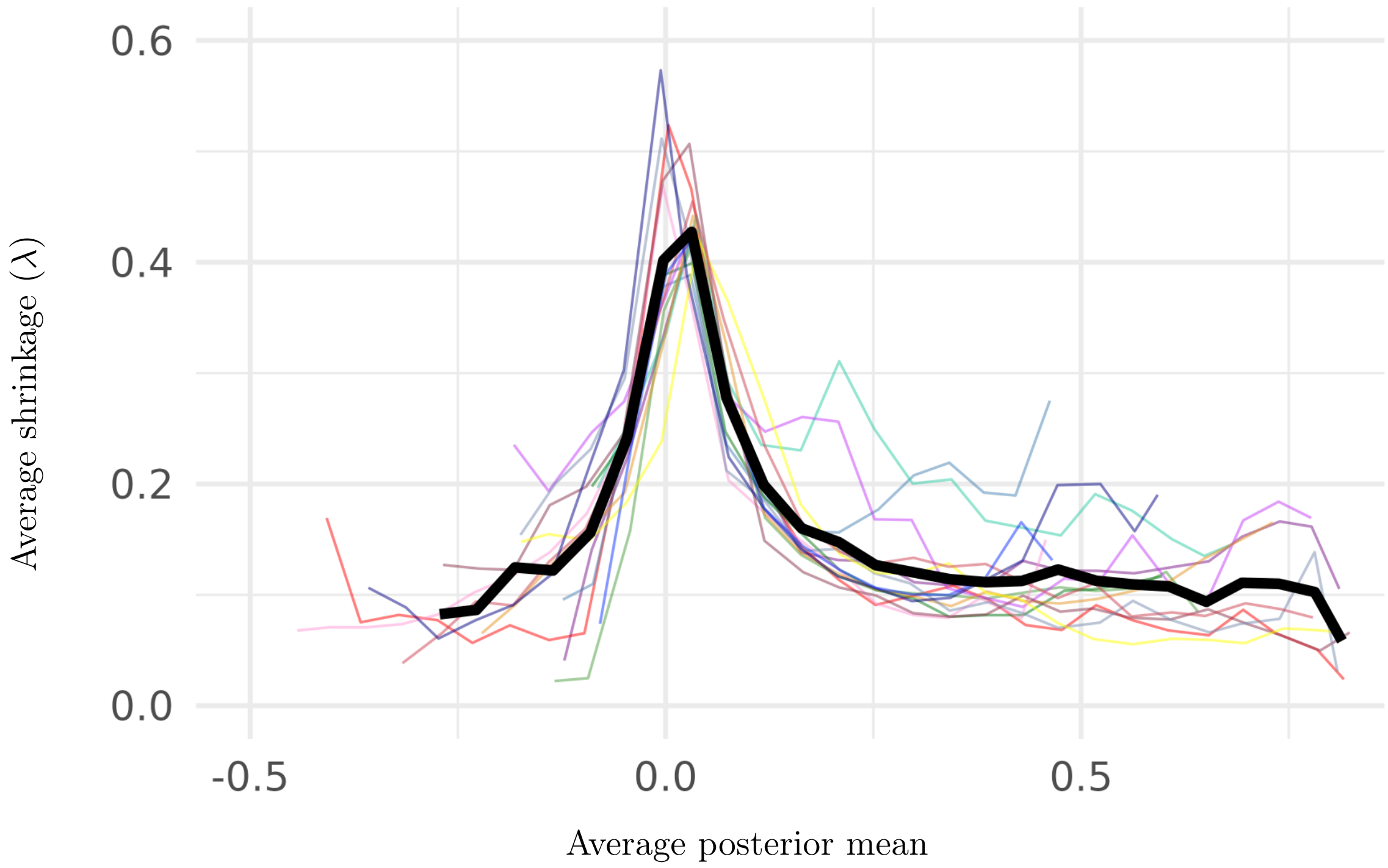

Figure S.3: Degree of shrinkage by posterior mean value. For 10 randomly selected HCP subjects, the posterior was calculated using the Yeo 17 priors, and a corresponding MLE was obtained by regressing the network time series against the BOLD data. All vertices were binned according to their posterior mean values, and then plotted against the average shrinkage of those same vertices. Each colored line represents a different network, while the black line represents the average shrinkage across components. The average is only displayed for posterior values represented in at least 5 networks. In background regions for a given network (posterior mean near zero), shrinkage is approximately 40-50%, but in regions with higher engagement shrinkage is approximately 10% on average.
